# Second trimester vaginal *Candida* colonization among pregnant women attending antenatal care in Bukavu, Democratic Republic of the Congo: prevalence, clinical correlates, risk factors and pregnancy outcomes

**DOI:** 10.3389/fgwh.2024.1339821

**Published:** 2024-05-23

**Authors:** Mulumeoderhwa Guy Mulinganya, Karen De Keyser, Irenge Jules Mongane, Mirindi Freddy Kampara, Annelies De Vulder, Jerina Boelens, Hans Duyvejonck, Erick Hendwa, Bisimwa Yvette Kujirakwinja, Balaluka Ghislain Bisimwa, Antonio Rodriguez, Mario Vaneechoutte, Steven Callens, Piet Cools

**Affiliations:** ^1^ Faculty of Medicine, Catholic University of Bukavu, Bukavu, Democratic Republic of the Congo; ^2^ Department of Obstetrics and Gynecology, Hôpital Provincial Général de Référence de Bukavu, Bukavu, Democratic Republic of the Congo; ^3^ Department of Internal Medicine and Pediatrics, Faculty of Medicine and Health Sciences, Ghent University, Ghent, Belgium; ^4^ Department of Diagnostic Sciences, Faculty of Medicine and Health Sciences, Ghent University, Ghent, Belgium; ^5^ Department of Laboratory Medicine, Ghent University Hospital, Ghent, Belgium

**Keywords:** candida, pregnancy, Democratic Republic of the Congo, newborn, preterm birth, qPCR, microscopy, vaginal microbiome

## Abstract

**Introduction:**

Vaginal *Candida* colonization (CC) can lead to vulvovaginal candidiasis, the second most prevalent vaginal condition worldwide, and has been associated with adverse birth outcomes. However, no data on CC in the Democratic Republic of the Congo are available. We investigated the prevalence, *Candida* species, clinical correlates, risk factors and pregnancy outcomes in women with CC in the second trimester of pregnancy.

**Material and methods:**

In Bukavu, the Democratic Republic of the Congo, pregnant women were recruited during antenatal care between 16 and 20 weeks of gestation from January 2017 to October 2017 and followed until delivery. Sociodemographics, sexual behavioral, hygienic and clinical characteristics, microbiological data and pregnancy outcomes were collected. *Candida* detection and speciation was performed with microscopy (Gram-stained smears and wet-mount) and/or quantitative PCR. Multivariate regression models were used to estimate the different associations with CC.

**Results:**

The prevalence of CC by wet mount, microscopy of Gram-stain smears and qPCR was 27.9%, 28.1% and 38.2%, respectively. *C. albicans* was the most prevalent *Candida* species (91.0%). Previous genital infections, an intermediate vaginal microbiota, bacterial vaginosis, and the use of pit toilets were risk factors for CC. Clinically, CC was associated with itching only. Women with CC had twice the odds for preterm birth, if *Candida* concentration was high, the odds were four times higher.

**Conclusions:**

In Bukavu, the Democratic Republic of the Congo, the prevalence of CC was high and associated with microbiological and modifiable risk factors. Screening and treatment for CC during antenatal care should be investigated as a possible strategy to reduce preterm birth.

## Introduction

A healthy vaginal microbiota (VMB) of women of reproductive age is typically dominated by only a single *Lactobacillus* species, mostly *L. crispatus*, *L. gasseri* or *L. jensenii* (1). The role of *L. iners* in reproductive health is under debate (2). These single *Lactobacillus* species or consortia of different *Lactobacillus* species (3) produce lactic acid by metabolizing the amylase breakdown products of glycogen present in the vagina. This acidifies the vagina and is a crucial first-line defense protecting women against urogenital tract infections. Furthermore, a healthy VMB is associated with healthy pregnancy outcomes. Vice versa, bacterial vaginosis (BV) is a dysbiosis of this healthy VMB predominated by anaerobes such as *Gardnerella* and *Fannyhessea* and linked with adverse pregnancy outcomes such as preterm birth, low birth weight and premature ruptures of membranes (1).

The yeast *Candida* can colonize the VMB as a commensal. This vaginal *Candida* colonization (CC) is more prevalent in case of elevated endogenous estrogen levels, such as during pregnancy (4). CC can transition from a commensal state to a pathogenic state—vulvovaginal candidiasis—under certain conditions that are incompletely understood (4, 5). Clinical therapy of vulvovaginal candidiasis is difficult due to the capacity of *Candida* to form biofilms (6) and withstand the host immune system (7).

In most populations, vulvovaginal candidiasis and bacterial vaginosis are the leading causes of vaginal dysbiosis in women of reproductive age (8). In by far most cases, vulvovaginal candidiasis is caused by *Candida albicans* (9), the remainder by non-albicans *Candida* spp., the most common of which is *Candida glabrata*. The usual presenting symptoms are acute pruritus and vaginal discharge, but none is specific (9). Vaginal discharge is present inconsistently and is often neglible, not uncommon symptoms include vaginal soreness, irritation, vulvar burning, dyspareunia (9). Importantly, asymptomatic CC be present in up to 50% of women (10). Besides clinical suspicion, microscopy has been used to diagnose vulvovaginal candidiasis for decades, however, culture has remained the gold standard (11).

In the Democratic Republic of the Congo (DRC), in case of absence of laboratory capacity, the syndromic management is implemented as recommended by the WHO (12). This syndromic management is based on the identification of consistent groups of symptoms and easily recognized signs, such as the vaginal discharge syndrome, and the treatment of the majority of, or the most serious, pathogens responsible for the syndrome (12). In practice, women presenting with vaginal discharge are often treated for BV, vulvovaginal candidiasis and trichomoniasis (13). This strategy results in frequent undertreatment or overtreatment, which is frequently accompanied by recurring vaginal problems.

In observational studies, vulvovaginal candidiasis but also CC have been conflictingly associated with increased risk of spontaneous preterm birth (PTB), the birth before 37 weeks of gestation (14, 15). PTB directly accounts for 35% of the annual neonatal mortality worldwide (16). A review approximated that 14.8 million infants were born preterm in 2014 with most cases occurring in Sub-Saharan Africa and Asia (17). In DRC, the neonatal mortality rate is estimated to be 2.8% (18) and 32% of these mortalities in DRC are caused by PTB complications (19).

Despite the importance of vulvovaginal candidiasis, and the potential detrimental role of CC in pregnancy, the latter is immensely understudied, especially in sub-Saharan Africa and DRC. There are no data on prevalence, *Candida* species distribution, clinical presentation, risk factors and the role of *Candida* in birth outcomes. Therefore, we aimed to address these major gaps in a population of pregnant women from Bukavu, DRC.

## Material and methods

### Study design and population

The current study was part of the *AVEONS* study, described previously (20). AVEONS was a prospective observational study and included women between 16 and 20 weeks of gestational age [visit 1 (V1)], between 36 and 38 weeks (V2) and at delivery. The main aim of AVEONS was to investigate the role of vaginal infections in adverse pregnancy outcomes in Bukavu, DRC. Newborns were followed from delivery until day seven of life. This manuscript only considers CC at V1 and pregnancy outcomes.

### Recruitment

Between January and October 2017, pregnant women were recruited when seeking antenatal care at the Provincial Referral Hospital of Bukavu (PRHB). Church announcements, radio and TV spots, community leader's meetings and posters were used to promote the study. Pregnant women who were interested in participating were informed individually and requested to sign an informed consent form. Participants were reimbursed for their transportation costs.

### Inclusion and exclusion criteria

Pregnant women were considered for inclusion if (i) they were between 16 and 20 weeks of gestation, (ii) they accepted to be followed by a referral hospital team and were willing to deliver at PRHB and (iii) they accepted to be contacted by phone or other means. Women were not considered eligible in case (i) they planned to move out of Bukavu during their pregnancy, (ii) of twin pregnancies, (iii) of a fetus with a visible malformation at ultrasound examination, (iv) they had genital bleeding and/or (v) of use of antibiotics during the 2 weeks before recruitment.

### Routine antenatal care and delivery procedures

A general physical and gynecological examination was performed. Urine (to diagnose urinary tract infections or bacteriuria) and five mL of total blood (to test for HIV, malaria and hemoglobin) was collected. Also, a gynecological examination, including a speculum examination with a sterile non-moistened speculum, was performed. During this examination, both the vaginal mucosa and cervix were inspected for the presence of sores and tumors. A vaginal infection was diagnosed according to the syndromic-based guidelines for the management of pregnancy issued by the Ministry of Public Health of DRC (21), which are based on WHO recommendations (22). This syndromic-based diagnosis was made in case the participant suffered from abnormal vaginal discharge (thick curdy), itching, redness, abnormal foul smell and/or burning sensation after intercourse. In case of vaginal infection, women were treated empirically with a combination of clotrimazole (200 mg) and clindamycin (100 mg). This combination is an efficient, safe and well-tolerated combination for treating mixed vaginitis caused by bacteria and fungi, also in pregnancy (23). Due to a lack of diagnostic tools in DRC, the syndromic method is employed to manage vulvovaginal diseases and a broad-spectrum combination medication capable of treating fungal, bacterial, and mixed infections is needed.

An ultrasound examination was performed to assess the viability of the fetus and to measure the cervical length. Women followed antenatal care as usual and were offered a single dose of 500 mg mebendazole (against intestinal worm infections) and a single dose of sulfadiazine-pyrimethamine (500 mg) against malaria. At delivery, pediatricians evaluated the newborns and assessed their anthropometric features. The WHO criteria were used to suspect an early newborn infection. Nurses and a senior assistant monitored labor and collected the relevant data, such as vital signs, vaginal delivery mode, obstetrical criteria of delivery, and symptoms of vulvovaginal infection.

### Study specific procedures

Data on sociodemographics and reproductive health history, sexual behavior, vaginal practices and complaints were obtained by questionnaires. The vaginal pH was determined by indicator pH papers (Hilindicator® pH paper). Three vaginal swabs and one cervicovaginal lavage (CVL) were taken. The first swab was rolled on three glass slides, one for direct wet mount microscopy and two for future Gram staining, the second swab was used for culturing and the third for molecular testing. CVLs were taken as previously described (24). Briefly, using a pipette, 10 ml of normal saline was flushed over the cervix and the lateral vaginal walls, aspirated as much as possible from the posterior fornix and collected in a vacutube. CVLs where then put in a cooled box (4 °C) and immediately transported to the laboratory, where they were frozen at −20 °C until shipment to the Laboratory Bacteriology Research (LBR) (Ghent University, Ghent, Belgium).

### Laboratory tests as part of the routine antenatal care

The serum was used to test for HIV (Alere Determine^TM^ HIV-1/2, Abbott, Diegem, Belgium), malaria (AG P.f/pan, Bioline®, Abbott, Diegem, Belgium) and hemoglobin (HemoCue Hb201+, HemoCue, Ängelholm, Sweden). The urine was tested for the presence of white blood cells and nitrite (indicating urinary tract infection or bacteriuria) by Multistix® dipsticks (Siemens, Groot-Bijgaarden, Belgium). The wet mounts were examined for the presence of clue cells (25), *Candida* and *Trichomonas vaginalis*.

### Study specific laboratory procedures

#### Microscopic detection of *Candida* and BV

The two vaginal smears were fixated, shipped to the LBR, Gram-stained using an automated Poly Stainer and scored by two independent readers according to the Nugent scoring system (26) to categorize the vaginal microbiota as healthy, intermediate microbiota or BV. In case of discrepancy in categorization, slides were reassessed by the two reviewers and discussed. In case no consensus was obtained, a third reader assessed the slide as a tie breaker. The presence of *Candida* (cells and/or hyphae) upon microscopic examination of Gram-stained smears was used as the case definition of CC.

#### Molecular detection and speciation of *Candida*

DNA was extracted from CVLs using the RNeasy PowerMicrobiome Kit (Qiagen, Antwerp, Belgium) according to manufacturer's instructions with minor modifications. To detect and speciate *Candida* in the CVL DNA extracts, fungal DNA was amplified in a qPCR assay using universal fungal primers targeting the internal transcribed spacer-2 region followed by high resolution melting of the amplicons and analysis of the melting curves for speciation as described previously (27). Briefly, a total reaction volume of 10 µl was prepared containing the primers ITS4 (5*'*-TCCTCCGCTTATTGATATGC-3*'*) and ITS86 (5*'*-GTGAATCATCGAATCTTTGAAC-3*'*), both at a final concentration of 0.5 μM, 1× LightCycler 480 HRM master mix (Roche) and two μl of DNA extract or control DNA. Amplification was carried out on a LightCycler 480 (Roche) by pre-incubating the reaction mixture for 10 min at 95 °C, followed by 45 cycles of 20 s at 95 °C, 30 s at 55 °C and 30 s at 72 °C. Hereafter, a high resolution melting curve was generated by first melting all DNA at 95 °C for 5 s, followed by renaturating DNA for 1 min at 60 °C, and gradually increasing the temperature from 60 °C to 97 °C at a ramp rate of 0.02 °C /s. Results were analyzed with the LightCycler 480 Software, version 1.5 (Roche), as described previously (27). As positive controls in each run, DNA from *Candida dubliniensis*, *C. famata*, *C. glabrata*, *C. guillermondi, C. inconspicua*, *C. kefyr*, *C. krusei*, *C. lipolytica*, *C. lusitania*, *C. metapsilosis*, *C. nivariensis*, *C. norvegensis*, *C. metapsilosis*, *C. ortopsilosis*, *C. parapsilosis*, *C. tropicalis* and *Saccharomyces cerevisiae* was used. In each run, ten negative template controls were used, and each sample was analyzed in duplicate. High resolution melting curves peaks of positive samples were compared with those of reference standards for identification of the yeast.

### Data analysis

A case of CC was defined based on the microscopic observation of *Candida* cells and/or hyphae on Gram-stained vaginal smears. PTB was defined as delivery before 37 completed weeks of gestation and after 28 completed weeks of gestation, low birth weight as birth weight <2,500 g (28–30).

For a comparison of the diagnostic performance of different assays, in case of absence of a true gold standard assay, a composite reference standard (CRS) can be used as the gold standard (31). For *Candida* detection, there is no gold standard, hence, a CRS for *Candida* colonization was used to assess sensitivity and specificity of the different *Candida* diagnostic methods. A case of *Candida* colonization using a CRS was defined if from the three diagnostic methods (wet mount microscopy, Gram-stain microscopy and qPCR) at least two of the assays were positive. This comparison of diagnostic performance of three methods was based on a subset of 330 samples. To investigate to which extent the sensitivity of Gram-stain and wet mount-based microscopy were function of the *Candida* concentration, we (i) stratified the qPCR positive samples into three more or less equal groups, i.e., low (<9.0E + 03 *Candida* cells/ml), moderate (9.0E + 03 *Candida* cells/ml—2.0E + 05 *Candida* cells/ml) and high concentration (>2.0E + 05 *Candida* cells/ml) and (ii) assessed the Gram-stain and wet mount positive rate in each of the concentration categories. Fleiss Cohen's kappa test was used to determine the agreement between *Candida* assays (32).

The Chi-squared test was used to compare CC with categorical independent variables. A binary logistic regression was performed to assess the associations between CC and different sociodemographic, sexual behavioral, hygienic, clinical, and anthropometric characteristics and laboratory findings (independent variables). These associations were expressed as crude odds ratios and 95% confidence interval (CI). Then, a multiple logistic regression model (using a combination of the stepwise forward or backward method) was applied to select CC risk factors and calculate adjusted odds ratios and 95% CI. This model was based on both probability-to-enter of 0.05 and probability-to-remove of 0.10.

Another multiple logistic regression model stratified by vaginal microbiota status was employed to estimate the association of CC with clinical symptoms. The goodness-of-fit of the final models was tested using the Hosmer-Lemeshow test.

A modified Poisson regression model with robust standard error was built to further investigate the extent to which CC was as an independent risk factor for adverse pregnancy outcomes [preterm birth (PTB), low birth weight, and premature ruptures of membranes (PROM)]. We corrected for *a priori* defined (possible) confounding factors influencing pregnancy outcome suggested by other studies (33–36) (vaginal microbiota, adjusted hemoglobin rate, cervix length, parity, body mass index, maternal age, education level and poverty).

All forms (laboratory forms, questionnaires, case reports forms) were scanned and stored digitally as a singular file. All data were entered by double data entry using the CSPRO software. The comparison data tool of CSPRO was used to compare the two data files (from the double data entry) for discrepancies. Discrepancies were resolved by consulting the original data in the scanned documents. Analyses were performed in STATA 14 (Stata Corp, College Station, US), *p*-values below 0.05 were considered as statistically significant.

### Ethics statement

This study was ethically approved by the Internal Review Board of the Catholic University of Bukavu (UCB/CIE/NC/016/2016), by the Ministry of Public health (062/CD/DPS/SK/2017) and by the Ethical Committee of Ghent University Hospital (PA2014/003). An informed consent form was signed by each pregnant woman who accepted to participate in the study.

## Results

### Sociodemographic description of the study population

**Figure 1:**
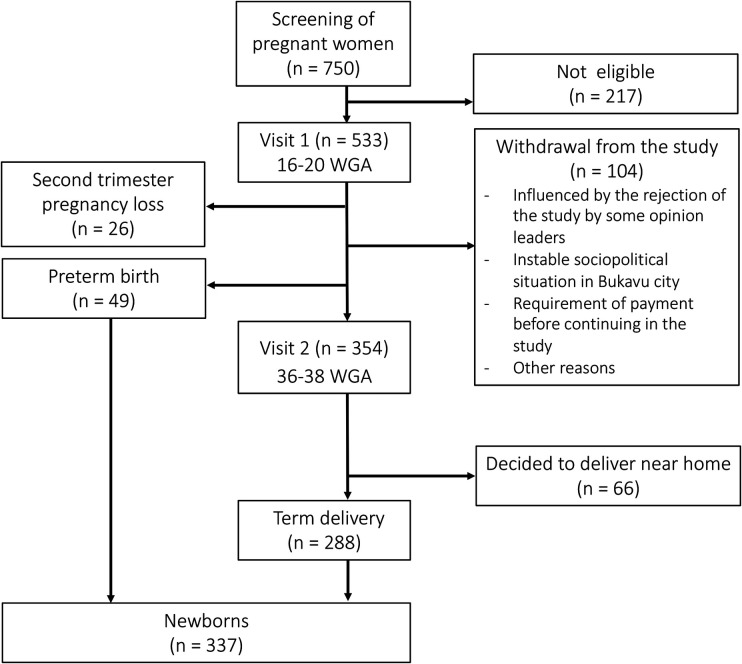
Flowchart of the study. WGA, weeks of gestational age.

The number of pregnant women and neonates withheld at each visit is shown in Figure 1.

A total of 750 pregnant women were screened of which 533 were found eligible and were enrolled in the cohort (V1). A total of 104 women (19.5%) who started in the study withdrew from the study before V2. A total of 26 (4.9%) women had a second trimester pregnancy loss. Of all newborns in the study, 288 (85.5%) were born at term and 49 (14.5%) were born preterm. Of the 354 women who completed V2, 66 (12.4%) decided to not deliver at PRHB but near their home.

The sociodemographic characteristics of the study population are outlined in Supplementary Information S1.

Near the half of pregnant women (48.4%) were symptomatic, i.e., suffered from an abnormal vaginal discharge, vaginal itching, burning sensation after sexual intercourse, and/or a foul smell from the vagina.

### Prevalence of vaginal *Candida* colonization and speciation

Table 1 documents the prevalence assessed in both symptomatic and asymptomatic pregnant women according to the different diagnostic tests. The overall prevalence of CC by means of microscopy of Gram-stained smears was 28.1%, in symptomatic and asymptomatic women, this was 48.1% and 8.3%, respectively. Using wet mount microscopy, the overall CC prevalence was 27.9%, in symptomatic and asymptomatic women, the prevalence was 55.6% and 1.2%, respectively. The overall CC prevalence assessed by qPCR (38.2%) was significantly higher compared to the microscopic methods, and similar in symptomatic and asymptomatic women (38.9% and 37.5%, respectively).

**Table 1:** Prevalence (with 95% confidence interval) of vaginal *Candida* colonization among symptomatic and asymptomatic pregnant women assessed by three diagnostic methods.

Diagnostic test	Total (*N* = 330)	Symptomatic women^a^ (*N* = 162)	Asymptomatic women (*N* = 168)
Gram-stained vaginal smears	28.1 (23.1–33.0)	48.1 (40.2–56.1)	8.3 (4.6–13.6)
Wet mount slides	27.9 (23.1–33.0)	55.6 (47.5–63.3)	1.2 (0.1–4.2)
qPCR	38.2 (32.9–43.7)	38.9 (31.3–46.8)	37.5 (30.1–45.3)

^a^ Defined as abnormal vaginal discharge, vaginal itching, burning sensation after sexual intercourse, and/or a foul smell from the vagina.

Among all women who carried *Candida* as assessed by means of qPCR, *Candida albicans* was the most prevalent species (91.0%) (Supplementary Information S2). The other yeast species were *C. famata* (1.6%), *C. glabrata* (0.8%), *C. dubliensis* (2.5%), *C. inconspicua* (0.8%), *C. kefyr* (0.8%), *C. krusei* (1.6%), *C. tropicalis* (0.8%) and *Sacharomyces cerevisiae* (3.2%).

### Diagnostic performance of microscopic and molecular tests

The diagnostic performance parameters are shown in Table 2. The highest sensitivity was found for qPCR (performed on CVLs) (99.0%), followed by microscopic examination of Gram-stained vaginal smears (82.9%) and wet mount preparations (76.2%). The specificity of the three assays was within the same range (90.5%–97.7%).

**Table 2:** Diagnostic performance (with 95% confidence interval) of the different tests for *Candida* vaginal colonization (wet mount, gram-stain, qPCR) based on 330 women.

	Wet mount	Gram-stain	qPCR
Kappa value^a^	0.74 (0.66–0.82)	0.74 (0.65–0.82)	0.85 (0.79–0.91)
Sensitivity	76.2 (66.9–84.0)	82.9 (74.3–89.5)	99.0 (94.8–100)
Specificity	95.5 (91.8–97.8)	97.7 (94.8–99.3)	90.5 (85.8–94.0)

^a^ For the comparison of the diagnostic performance of the different *Candida* assays (i.e., wet mount microscopy, Gram-stain microscopy, and qPCR), a composite reference standard (CRS) was considered as the gold standard and a positive CC case was defined if at least two of these assays were positive.

In comparison with the CRS, a good agreement was observed with both the wet mount and Gram-stain-based microscopy (*κ* = 0.74 (0.66–0.82) and 0.74 (0.65–0.82), respectively) and an almost perfect agreement with qPCR [*κ* = 0.85 (0.79–0.91)].

**Figure 2:**
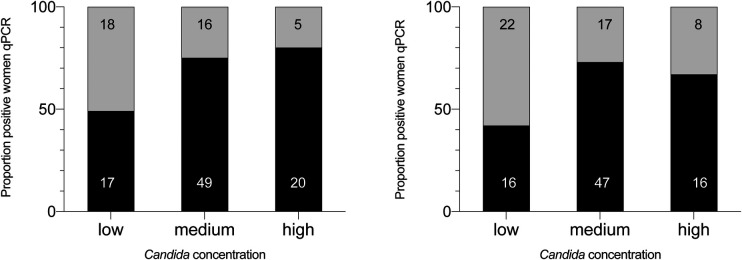
Positivity rate of vaginal *Candida* colonization as assessed by microscopic techniques according to *Candida* concentration measured by qPCR. The different classes of *Candida* concentration (assessed by means of qPCR) are categorized into low, moderate and high. The black part of each column represents the proportion of samples found positive by Gram-stain or wet mount in the different categories, the grey part the samples found negative. The number in the columns are absolute numbers, the numbers on the Y-axis are percentages. GS, microscopic examination of Gram-stained smears; WM, microscopic examination of wet mounts.

The positivity rate of the two microscopic techniques as function of the *Candida* concentration (as assessed by means of qPCR) is shown in Figure 2. The positivity rate of microscopic techniques increased with increasing concentration, with the highest microscopic detection rate for Gram stain microscopy (80%) in the *high concentration* group (>2E05 *Candida* cells/ml).

### Independent risk factors for vaginal *Candida* colonization

The univariate and multivariate associations are shown in Supplementary Information S1 and Table 3, respectively. CC was significantly and independently associated with the use of a pit toilet compared to a flush toilet (aOR: 1.85; 95% CI: 1.22–2.81), a history of vaginal infection in the six previous months (aOR: 4.90; 95% CI: 1.37–17.43), the practice of labia elongation (aOR: 0.46; 95% CI: 0.25–0.84), the presence of an intermediate vaginal microbiota (aOR: 2.87; 95% CI: 1.73–4.77) and BV (aOR: 2.27; 95% CI: 1.42–2.81).

**Table 3:** Multivariate risk factor associations with vaginal *Candida* colonization.

Characteristics	CC + *n* (%)	cOR (95%CI)	aOR (95% CI)	*p*-value
Type of toilet				
Flushing toilet	49 (21.9)	Ref.	Ref.	
Pit toilet	99 (32.9)	**1.75 (1.18–2.61)**	**1.85 (1.22–2.81)**	**0**.**004**
Mode of cleaning perineum after defecation				
Water	109 (31.1)	Ref.	Ref.	
Tissue paper/other materials	39 (22.4)	**0.64 (0.42–0.98)**	0.66 (0.42–0.1.03)	0.069
Duration of marriage				
≤5 years	89 (30.9)	Ref.	Ref.	
>5 years	59 (24.9)	0.74 (0.50–1.09)	**0.65 (0.43–0.98)**	**0.042**
History of vaginal infection^a^				
No	141 (27.5)	Ref.	Ref.	
Yes	7 (58.3)	**3.69 (1.15–11.83)**	**4.90 (1.37–17.43)**	**0**.**014**
Labia elongation^b^				
No	30.6	Ref.	Ref.	
Yes	17.2	**0.47 (0.26–0.84)**	**0.46 (0.25–0.84)**	**0**.**012**
Vaginal microbiota				
Healthy vaginal microbiota	57 (20.0)	Ref.	Ref.	
Intermediate vaginal microbiota	42 (41.2)	**2.80 (1.72–4.57)**	**2.87 (1.73–4.77)**	**<0**.**001**
Bacterial vaginosis	49 (35.5)	**2.20 (1.40–3.47)**	**2.27 (1.42–2.81)**	**0**.**001**

aOR, adjusted odds ratio; CC, vaginal *Candida* colonization; cOR, crude odds ratio; Ref., reference category; 95% CI, 95% confidence interval.

Bold values represent values that are statistically significant at the *P* < 0.05 level.

^a^ In the last six months.

^b^ An old practice to lengthen outer lips by using herbs during adolescence.

### Symptomatology of vaginal *Candida* colonization

The univariate associations of clinical signs and symptoms with CC are shown in Supplementary Information S1. The multivariate model stratified by the VMB status is shown in Table 4. Vaginal itching was statistically significantly associated with CC across strata (aOR: 1.98, 95% CI: 1.02–3.83; aOR: 3.26, 95% CI: 1.30–8.17; aOR: 3.40, 95% CI: 1.50–7.70 for healthy VMB, intermediate VMB, or bacterial vaginosis, respectively).

**Table 4:** Clinical symptoms associated with vaginal *Candida* colonization stratified by the vaginal microbiota status.

	Healthy microbiota		Intermediate microbiota		Bacterial vaginosis	
	aOR (95% CI)	*P*-value	aOR (95% CI)	*P*-value	aOR (95% CI)	*P*-value
Discharge		0.067		0.587		**0.049**
No	Ref.		Ref.		Ref.	
Yes	1.83 (0.96–3.50)		1.30 (0.51–3.27)		**2.24 (1.00–5.00)**	
Vaginal itching		**0.042**		**0.011**		**0.003**
No	Ref.		Ref.		Ref.	
Yes	**1.98 (1.02–3.83)**		**3.26 (1.30–8.17)**		**3.40 (1.50–7.70)**	
Burning sensation		0.066		0.565		0.096
No	Ref.		Ref.		Ref.	
Yes	1.83 (0.96–3.52)		1.33 (0.50–3.60)		0.48 (0.21–1.14)	
Vaginal foul smell		0.520		0.246		0.633
No	Ref.		Ref.		Ref.	
Yes	1.26 (0.61–2.62)		1.90 (0.64–5.50)		0.80 (0.32–1.997)	

aOR, adjusted odds ratio; Ref., reference category; 95% CI, 95% confidence interval.

Bold values represent values that are statistically significant at the *P* < 0.05 level.

### The univariate and multivariate associations of *Candida* with adverse pregnancy outcomes

The univariate associations between CC and delivery characteristics and neonatal outcomes are shown in Supplementary Information S3. Statistically significant associations were found between CC and PTB (OR: 1.59; 95% CI: 1.06–2.39), non-cephalic presentation (compared to cephalic presentation) (OR: 5.05; 95% CI: 1.44–17.72), neonatal fever (OR: 2.84; 95% CI: 1.56–5.15) and an elevated neonatal temperature (>37.2 °C, compared to 36.6 °C-37.2 °C) (OR: 2.09; 95% CI: 1.23–3.55). In the multivariate model (Table 5), CC remained significantly associated only with PTB (aOR: 1.94; 95% CI: 1.14–3.30, *p* = 0.014).

**Table 5:** Multiple regression model for preterm birth and vaginal *Candida* colonization.

Characteristics	aOR (95% CI)	*P*-value
Vaginal *Candida* colonization		
Absent	1	
Present	**1.94** (**1.14–3.30)**	**0**.**014**
Vaginal microbiota^a^		
Healthy vaginal microbiota	1	
Intermediate vaginal microbiota	0.83 (0.42–1.63)	0.587
Bacterial vaginosis	1.05 (0.58–1.92)	0.861
Hemoglobin		
Normal	1	
Anemia	1.45 (0.79–2.66)	0.224
Cervix length		
≥30 mm	1	
<30 mm	1.65 (0.81–3.53)	0.164
Parity	0.88 (0.73–1.05)	0.170
Poverty		
Not poor	1	
Poor	1.14 (0.60–2.13)	0.690
Education		
Primary	1	
Secondary	0.77 (0.29–2.05)	0.610
Higher	1.03 (0.37–2.86)	0.947
Age	1.07 (0.99–1.08)	0.065

aOR, adjusted odds ratio.

Bold values represent values that are statistically significant at the *P* < 0.05 level.

^a^ As assessed by means of the Nugent score.

The prevalence of PTB further stratified by *Candida* concentration is shown in Supplementary Information S4. There was an increasing prevalence of PTB, going from 11.2% (no *Candida* group), 16.0% (women with low *Candida* concentration), 19.4% (moderate concentration) and 31.3% (high concentration). Only high concentrations of *Candida* were found to be statistically significantly associated with PTB (OR, 3.60; 95% CI, 1.09–11.90; *p* = 0.035).

## Discussion

We aimed to investigate the prevalence, species, risk factor and clinical correlates of CC, as well as the association with pregnancy outcomes. To be best of our knowledge, no studies on *Candida* in pregnancy have been performed in DRC. The prevalence of CC was high, and associated with other genital tract infections, hygiene, and preterm birth.

### The prevalence of vaginal *Candida* colonization in pregnant women from Bukavu was high

We found a prevalence of 28.1% of CC in second trimester of pregnancy, as assessed by microscopy of Gram-stained smears; wet mount gave very similar results. Not surprisingly, in our study, the prevalence of CC as assessed by qPCR was much higher (38%). Interestingly, there was a clear difference in prevalence between symptomatic (48% and 56% for Gram-stain and wet mount, respectively) and asymptomatic women (8% and 1% for Gram-stain and wet mount, respectively) for the microscopic methods only. Such as difference was not seen for the qPCR method (symptomatic and asymptomatic women 39% and 38%, respectively). This might be due to the higher sensitivity of qPCR. The concentration of *Candida* is likely higher when the patient is symptomatic and it is also possible that qPCR is detecting DNA from dead/lysed *Candida* cells, hence, not visible by microscopy.

An overview of studies from other countries in sub-Saharan Africa reporting CC rates in pregnant women is given in Supplementary Information S5. In Sub-Saharan Africa in pregnancy, prevalence ranges from 14% (Burkina Faso) to 57% (South Africa) in the normal pregnant population. Differences in prevalence between all studies may be explained by different populations and the use of different diagnostic tests, which have different sensitivities, as has been shown in current study and by others (37–39). Also, the different mean gestational age between populations can impact *Candida* prevalence (36), although some studies from sub-Saharan Africa found no statistically significant association with gestational age (10, 40, 41).

We found that *C. albicans*—the most virulent *Candida* species colonizing the vagina (42–44)—was the most prevalent yeast (91.0%). Other studies from sub-Saharan Africa documented *C. albicans* as the most yeast species in pregnant women, ranging from 40.4% to 90.7% (Supplementary Information S3). The non-albicans species (*C. glabrata*, *C. dubliensis*, *C. krusei*, and *C. tropicalis)* in our study all have been isolated previously from cases of vaginitis, with *C. glabrata* being considered the most prevalent cause of non-albicans vaginitis. Other species found in our study, *C. famata*, *C. inconspicua*, *C. kefyr* and *S. cerevisiae*, are less common to (very) rare causes of vaginitis (45, 46).

### Previous vaginal infections and concurrent VM disturbances were independent risk factors for vaginal *Candida* colonization

Women with a history of vaginal infection in the last six months had a nearly five-time higher odds of *Candida* colonization. Although we have no specific information on the type of these previous vaginal infections, not unlikely, these were (i) (recurrent) vulvovaginal candidiasis, which has a global prevalence of 9% in the age group 25–34 years (47), (ii) and/or a previous STI, a risk factor for vulvovaginal candidiasis (48), (iii) and/or (recurrent) intermediate VM/BV, which were also risk factors for CC in our study. Indeed, women with an intermediate VMB or BV had a two- to three-fold higher odds for CC. The protective role of (probiotic) lactobacilli against *Candida albicans* is controversial (49) and clinical studies have shown conflicting findings on the association between the VM and vaginal *Candida* (50, 51). It has been demonstrated different *Lactobacillus* species have different probiotic effects against *Candida in vitro* (52).

### Improved sanitation might reduce the prevalence of vaginal *Candida* colonization

Women using a pit toilet had a higher risk for CC compared to women using a flushing toilet. Commonly, pit toilets lack (sufficient) running water and/or soap for a proper cleaning of hands and/or perineum. As the gut has been documented as a reservoir for vaginal *Candida* (53), poor cleansing could enhance the spread *Candida* from perineum to the vagina and everywhere hand-touched in the toilet. Pit toilets have also been associated with other urogenital infections such as trichomoniasis (54, 55). Our findings further stress the need of improving sanitation facilities—also in urban areas such as in our study—to reduce urogenital infections, especially among pregnant women.

We further found that pregnant women who practiced labia elongation had a lower risk of CC. In several central African countries, lengthening of the labia is done as an esthetic improvement, to increase sexual pleasure, and/or to be accepted by the tribe (56).

### Vaginal *Candida* colonization was independently associated with preterm birth

Prevention of PTB remains one of the most important challenges in maternity and neonatology care (16, 17). Although the etiology of PTB is multifactorial, both local and systemic infections are a major cause (57).

Here, we found that CC during the second trimester of pregnancy was independently associated with PTB, with women who carried *Candida* having a two-fold higher odds for PTB. Importantly, these odds were nearly four-fold in women colonized with high concentrations of *Candida*, defined in our study as >2E05 *Candida* cells/ml by means of qPCR.

A high concentration of *Candida* might increase the possibility of an ascending infection leading to PTB. In some cases, vaginal pathogen concentrations are indeed important in reproductively health. For example, Goodfellow and colleagues showed that the vaginal concentration of *L. iners* was associated with early PTB recurrence (58). Also, in Group B *Streptococcus* (GBS) neonatal sepsis, GBS is transmitted from the maternal vagina to the neonate during birth or after breaking of the fetal membranes. It is known that there is a direct relationship between the maternal vaginal GBS concentration, the risk of vertical transmission and the likelihood of serious disease in neonates (59).

Future studies investigating the role of the VMB in pregnancy should consider absolute concentrations. This is currently a shortcoming of the widely used 16S rRNA gene metagenomic next-generation sequencing studies that report in relative abundances. Himschoot et al. (2024, under revision) showed that across samples, the same relative abundances of vaginal key species can represent a large range (several logs) of absolute concentrations.

Two meta-analyses (one considering only asymptomatic CC (14) and one considering both symptomatic and asymptomatic CC (15)) reported no association between (asymptomatic) vaginal *Candida* and PTB. Several factors might explain why our findings are contrasting.

First, only two studies (from Kenya) in these meta-analyses were performed in sub-Saharan Africa. Both PTB and CC, common among black women, are influenced by maternal ethnicity (60, 61). Also, the VMB differs between African and Caucasian populations (62). Furthermore, there is an important difference in the management (including treatment) of vaginal *Candida* between high-income countries and low-and-middle income countries (see further), and treatment differences between studies are present. Second, we tested for CC in the second trimester of pregnancy, but the timepoint(s) of testing varied in other studies. *Candida* later in pregnancy might have less impact on PTB. Third, diagnostic methods differ between studies.

### *Candida* and BV management in antenatal care

In DRC, self-reported vaginal symptoms remain the backbone of the syndromic management of genital infections, and clinical examination or microscopy are not often used. This approach has been shown to perform poorly, for example, an evaluation in Kenya showed that only 50% of women presenting with vaginal complaints received appropriate treatment (63). Currently, the WHO guidelines for the management of genital infections have included recommendations for the use of molecular assays or more simple point-of-care tests, but implementing this remains a challenge/unfeasible in most resource-limited settings. We showed that microscopic examination of wet mount preparations and/or Gram-stained smears—even with a sub-optimal sensitivity compared to qPCR—only fails to detect *Candida* if present at low concentrations (as assessed by qPCR), which might be clinically less relevant and was not associated with PTB in our study.

In our study, 45% of women had CC and/or BV (19% CC, 17% BV and 9% CC/BV coinfection). Often, symptoms of these conditions are similar, complicating clinical diagnosis and effective treatment (47, 64). In our study, the only symptom associated with CC across VMB categories of healthy (OR 1.98), intermediate (3.26) and BV (OR 3.40) was vaginal itching, similar to findings reported by Gloria and colleagues (OR 2.20, 95% CI 1.40–3.46) (63).

WHO recommends to screen women with previous spontaneous abortion, stillbirth and/or preterm delivery for BV and trichomoniasis (65), and our findings call for further investigation if *Candida* should be included. Microscopy could be considered as routine screening of women in antenatal care. Although microscopy is an affordable and rather easy to implement technique, it's use often seems to be arduous and tedious owing to the lack of a relevant practitioner's training. Development and implementation of cheap and quick point-of-care tests should be intensified as a means for the reduction of PTB in resources constrained countries. These assays should include multiple targets (*Candida*, BV, and *Trichomonas vaginalis*).

### Limitations and future perspectives

Our study was limited by the drop-out of approximately one third of the study participants during follow-up due to socio-economic and political reasons. Because of this, our study might be underpowered to assess the association between CC and PTB, hence, results should be interpreted with care.

Considering we found a substantial part of pregnant women, not only from our study but also other Sub-Saharan African countries (see Supplementary Information S3), were colonized by *Candida*, and CC was associated with increased risk for PTB, further research should be done. A meta-analysis of two randomized controlled trials indicated that clotrimazole treatment for vaginal *Candida* reduced the risk for spontaneous preterm birth compared to standard of care (risk ratio 0.36, 95% CI 0.17–0.75) (60). Similar trials should investigate if a similar approach also can reduce the incidence of PTB in DRC. To guide such interventions, research on the development and implementation of cheap point-of-care tests and/or microscopy to diagnose *Candida*, BV and sexually transmitted infections in antenatal care in LMIC should be intensified.

## Conclusion

In this first report on vaginal *Candida* in women from DRC, we document a high prevalence of CC in pregnancy, mainly *C. albicans*. CC was associated with microbiological and modifiable risk factors. Vaginal *Candida*, especially when present at high concentrations, was an independent risk factor for PTB.

## Data Availability

The raw data supporting the conclusions of this article will be made available by the authors upon request, without undue reservation.

## Key message

Vaginal *Candida* colonization is associated with preterm birth in a population from the Democratic Republic of the Congo.
