# Supplementary material for: Second trimester vaginal Candida colonization among pregnant women attending antenatal care in Bukavu, Democratic Republic of the Congo: prevalence, clinical correlates, risk factors and pregnancy outcomes
Source: Front Glob Womens Health. 2024 May 23;5:1339821. doi: 10.3389/fgwh.2024.1339821 (PMC11153668; doi:10.3389/fgwh.2024.1339821)
Supplement: Supplementary file 1 [file Table1.pdf]

SUPPLEMENTARY INFORMATION

Supplementary Information 1: Univariate associations of sociodemographic, sexual behavior, hygienic and clinical characteristics, laboratory findings with vaginal *Candida* colonization (CC) (N=525[a])

	n (%)	CC+ (%)	COR (95%CI)	p-value
Sociodemographic characteristics				
**Age (years)**				0.792
<20 years	26 (5.0)	30.8	1.19 (0.49–2.92)	
20-24	113 (21.5)	30.1	1.15 (0.68–1.95)	
25 –29	173 (33.0)	27.2	Ref.	
30–34	135 (25.7)	30.4	1.17 (0.71–1.92)	
>35	78 (14.9)	23.1	0.80 (0.43–1.50)	
**Tribe[(b)]**				0.743
Shi	374 (71.2)	29.1	Ref.	
Rega	57 (10.9)	26.3	0.87 (0.46–1.63)	
Other tribes	94 (17.9)	25.5	0.83 (0.50–1.39)	
**Religion[(c)]**				0.781
Christian	492 (93.7)	28.1	Ref.	
Not Christian	33 (6.3)	30.3	1.12 (0.52–2.40)	
**Quality of life[(d)]**				0.063
Not poor	144 (27.4)	22.2	Ref.	
Poor	381 (72.6)	30.5	1.53 (0.98–2.40)	
**Employment status**				0.778
Employed or self-employed	89 (17.0)	27.0	Ref.	
Unemployed	436 (83.0)	28.4	1.08 (0.64–1.80)	
**Marital status**				0.567
Married	501 (95.4)	27.9	Ref.	
Unmarried	24 (4.6)	33.3	1.29 (0.54–3.08)	
**Education[(e)]**				0.477
Higher	190 (36.2)	25.3	Ref.	
Primary	62 (11.8)	27.4	1.12 (0.59–2.13)	
Secondary	273 (52.0)	30.4	1.29 (0.85–1.96)	
**Alcohol consumption[(f)]**				0.273
No	335 (63.8)	26.6	Ref.	
Yes	190 (36.2)	31.1	1.24 (0.84–1.84)	
**Clay consumption[(g)]**				0.870
No	384 (73.1)	28.4	Ref.	
Yes	141 (26.9)	27.7	0.96 (0.63–1.48)	
**Tobacco use[(h)]**				0.508

No	523 (99.6)	28.1	Ref.	
Yes	2 (0.4)	50.0	2.56 (0.16–41.16)	
Sexual behaviour characteristics				
**Age at marriage**				0.687
>18 years	407 (77.5)	27.8	Ref.	
≤18 years	118 (22.3)	29.7	1.10 (0.70–1.72)	
**Duration of marriage**				0.128
≤5 years	288 (54.9)	30.9	Ref.	
>5 years	237 (45.1)	24.9	0.74 (0.50–1.09)	
**First sexual intercourse**				0.675
≥ 18 years	369 (70.3)	28.7	Ref.	
<18 years	156 (29.7)	26.9	0.91 (0.60–1.39)	
**Anal intercourse practice**				0.280
No	473 (90.1)	27.5	Ref.	
Yes	52 (9.9)	34.6	1.40 (0.76–2.56)	
**Labia elongation**[(i)]				0.011
No	432 (82.3)	30.6	Ref.	
Yes	93 (17.7)	17.2	**0.47 (0.26–0.84)**	
**Extra sexual partners concurrent pregnancy**[(j)]				0.564
No	515 (98.1)	28.4	Ref.	
Yes	10 (1.9)	20.0	0.63 (0.13–3.01)	
**Circumcision of husband**				0.968
Circumcised	507 (96.6)	28.2	Ref.	
Uncircumcised	18 (3.4)	27.8	0.98 (0.34–2.80)	
**Concurrent husband's extra partners**[(k)]				0.838
No	474 (90.3)	28.1	Ref.	
Yes	51 (9.7)	29.4	1.07 (0.57–2.02)	
Sanitation and hygiene characteristics				
**Type of toilet**				0.006
Flushing toilet	224 (42.7)	21.9	Ref.	
Pit toilet	301 (57.3)	32.9	**1.75 (1.18–2.61)**	
**Mode of cleaning perineum after toilet**				0.039
Water	351 (66.7)	31.1	Ref.	
Tissue paper/other materials	174 (33.1)	22.4	**0.64 (0.42–0.98)**	
**Substances used during intimate toilet**				0.208
Water only	426 (81.1)	27.0	Ref.	
Varied substances [(l)]	99 (18.9)	33.3	1.35 (0.85–2.16)	
**Number of intimae toilets per day**[(m)]				0.651
≤2 per day	292 (55.6)	72.6	Ref.	

>2 per day	233 (44.4)	70.8	1.09 (0.75–1.60)	
Obstetrical characteristics				
**Parity**				0.227
0	137 (26.1)	26.3	1.03 (0.63–1.68)	
1	81 (15.4)	28.4	1.15 (0.65–2.03)	
2	89 (17.0)	37.1	**1.70 (1.01–2.89)**	
≥3	218 (41.5)	25.7	Ref.	
**Previous PTB**	**527**			0.983
No	500 (95.2)	28.2	Ref.	
Yes	25 (4.8)	28.0	0.99 (0.40–2.42)	
**Previous abortion**				0.420
No	425 (98.4)	28.7	Ref.	
Yes	7 (1.6)	42.9	1.86 (0.41–8.45)	
**History of vaginal infection**[n]				0.028
No	513 (97.7)	27.5	Ref.	
Yes	12 (2.3)	58.3	**3.69 (1.15–11.83)**	
Clinical, and anthropometrics findings				
**Clinical status at first visit**				<0.001
Asymptomatic	271 (51.6)	11.1	Ref.	
Symptomatic	254 (48.4)	46.5	**6.97 (4.43–10.96)**	
**Current abnormal vaginal discharge**				<0.001
No	274 (52.2)	18.6	Ref.	
Yes	251 (47.8)	38.7	**2.75 (1.85–4.09)**	
**Current vaginal itching**				<0.001
No	310 (59.0)	17.7	Ref.	
Yes	215 (41.0)	43.3	**3.53 (2.38–5.26)**	
**Current dysuria**				0.239
No	385 (73.3)	26.9	Ref.	
Yes	140 (26.7)	32.1	1.29 (0.85–1.96)	
**Burning sensation after sexual intercourse**				0.002
No	354 (67.4)	24.0	Ref.	
Yes	171 (32.6)	36.8	**1.85 (1.24–2.74)**	
**Current vaginal foul smell**				0.011
No	411 (78.3)	25.6	Ref.	
Yes	114 (21.7)	37.7	**1.76 (1.14–2.74)**	

**Cervix length**				0.590
>30 mm	473 (90.1)	28.5	Ref.	
≤30 mm	52 (9.9)	25.0	0.83 (0.43–1.61)	
**Maternal high blood pressure[(o)]**				0.685
No	520 (99.0)	28.3	Ref.	
Yes	5 (1.0)	20.0	0.63 (0.07–5.72)	
**Body mass index[(p)]**				0.141
Lean	219 (41.7)	32.4	Ref.	
Overweight	199 (37.9)	23.1	**0.63 (0.41–0.97)**	
Obesity class I	83 (15.8)	31.3	0.95 (0.55–1.64)	
Obesity class II & III	24 (4.8)	20.8	0.55 (0.20–1.53)	
**MUAC at recruitment[(q)]**				0.902
≥ 22 cm	474 (90.3)	28.3	Ref.	
<22 cm	51 (9.7)	27.5	0.96 (0.50–1.83)	
Laboratory findings				
**Nugent score**				<0.001
Healthy microbiota (0–3)	285 (54.3)	20.0	Ref.	
Intermediate microbiota (4–7)	102 (19.4)	41.2	**2.80 (1.72–4.57)**	
Bacterial vaginosis (8–10)	138 (26.3)	35.5	**2.20 (1.40–3.47)**	
**Vaginal pH**				0.531
≤4.5	8 (1.5)	25.0	Ref.	
4.6–6	406 (77.3)	27.1	1.11 (0.22–5.61)	
>6	111 (21.1)	32.4	1.44 (0.28–7.49)	
***Trichomonas* on wet mount**				0.393
No	518 (98.7)	28.0	Ref.	
Yes	7 (1.3)	42.9	1.93 (0.43–8.73)	
***Candida* on wet mount**				<0.001
No	387 (73.7)	15.3	Ref.	
Yes	138 (26.3)	64.5	**10.10 (6.47–15.76)**	
**Adjusted maternal haemoglobin[(r)]**				0.819
≥ 110 g/l	408 (77.7)	28.4	Ref.	
< 110 g/l	117 (22.3)	27.4	0.95 (0.60–1.50)	
**Malaria rapid test[(s)]**				0.508
Negative	523 (99.6)	28.1	Ref.	
Positive	2 (0.4)	50.0	2.56 (0.16–41.16)	
**HIV rapid test[(t)]**				
Negative	523 (99.6)	27.9	Ref.	
Positive	2 (0.4)	100.0	N/A	

Prophylaxis and treatment				
**Use of mosquito net**				0.695
Yes	449 (85.5)	28.5	Ref.	
No	76 (14.5)	26.3	1.12 (0.64–1.94)	
**Chemoprophylaxis against malaria[(u)]**				0.324
No	437 (83.2)	29.1	Ref.	
Yes	88 (16.7)	23.9	0.77 (0.45–1.30)	
**Chemoprophylaxis against worms[(v)]**				0.926
No	427 (81.3)	28.1	Ref.	
Yes	98 (18.7)	28.6	1.02 (0.63–1.66)	

(a) Eight slides did not contain biological material or appeared damaged.

(b) Other tribes (Tembo, Fuliru, Hunde, Nyanga, Hutu, Nande, Vira, Bembe) each <2.5%

(c) Christian represents Catholics, Protestants, Anglicans, Kimbanguistes and members of Revival Church Participants; Not Christian represents Muslim, Animist, and nonbelievers' participants

(d) Taking into account local parameters, poverty was calculated considering the type of the floor, water source, electricity, commodities in the house (We did not include income because it is very unstable and depends mainly on the informal sector). The total score ranged from 4 to 17. A score < 10, living under the threshold of poverty; a score ≥10, living above the threshold of poverty.

(e) Each educational levels contained participants who completed and not fully completed the level.

(f) More or equal to one or two glasses of 30 cl per day of local beer (4–5.5% alcohol content)

(g) More or equal to a regular basis consumption during current pregnancy (approximately 20 gram, 5 times a day)

(h) Tobacco use: use of cigarettes and/or chewing tobacco

(i) Labia elongation: an old practice to lengthen outer lips by using herbs during adolescence

(j) Concurrent pregnant woman's extra partners: male sexual partnerships of the pregnant women in the last six months that overlap in time as opposed to running sequentially(Kenyon & Osbak, 2014)

(k) Known female sexual partnerships of the husband of the pregnant women that overlap in time as opposed to running sequentially (Kenyon & Osbak, 2014)

(l) Soap, herbs, mixed powders, lemon, disinfectant products (Dettol[R] or benzalkonium chloride)

(m) Intimate toilet is defined as the process of intravaginal cleansing with liquid solution.

(n) Presence of vaginal infection the last six months

(o) Systolic pressure > 140 mm Hg and/or diastolic pressure >90 mm Hg

(p) Lean, combination of underweight category (≤18.5 kg/m^2) (only 8 participants) and normal range category (18.5–24.9 kg/m^2); class II & III obese is the combination of severe obesity (35.0–39.9. kg/m^2) class and very severe obesity (≥40.0 kg/m^2).

(q) Mid-Upper Arm circumference; measured for early detection of malnutrition

(r) Hemoglobin adjusted to the altitude of Bukavu (1498m) by reducing 20 grams per liter (Organization, 2011)

(s) SD BIOLINE malaria Ag P. f/Pan, a qualitative and differential test for the detection of histidine-rich protein II antigen of *Plasmodium falciparum* and common plasmodium lactate dehydrogenase (p LDH) of plasmodium species in human whole blood

(t) Determine HIV rapid test ALERE HIV1/2

(u) Sulfadoxine-pyrimethamine

(v) Mebendazole
