# Supplementary material for: Second trimester vaginal Candida colonization among pregnant women attending antenatal care in Bukavu, Democratic Republic of the Congo: prevalence, clinical correlates, risk factors and pregnancy outcomes
Source: Front Glob Womens Health. 2024 May 23;5:1339821. doi: 10.3389/fgwh.2024.1339821 (PMC11153668; doi:10.3389/fgwh.2024.1339821)
Supplement: Supplementary file 2 [file Table2.pdf]

Supplementary Information 2. **Speciation of *Candida* as assessed by qPCR followed by high resolution melting curve analysis.**

**Species**		**N = 122**
Candida albicans		111 (91.0%)
Non-albicans *Candida* species		11 (9.0%)
	C. famata	2 (1.6%)
	C. glabrata	1 (0.8%)
	C. dubliensis	3 (2.5%)
	C. inconspicua	1 (0.8%)
	C. kefyr	1 (0.8%)
	C. krusei	2 (1.6%)
	C. tropicalis	1 (0.8%)
Sacharomyces cerevisiae		4 (3.2%)
