# Supplementary material for: Second trimester vaginal Candida colonization among pregnant women attending antenatal care in Bukavu, Democratic Republic of the Congo: prevalence, clinical correlates, risk factors and pregnancy outcomes
Source: Front Glob Womens Health. 2024 May 23;5:1339821. doi: 10.3389/fgwh.2024.1339821 (PMC11153668; doi:10.3389/fgwh.2024.1339821)
Supplement: Supplementary file 3 [file Table3.pdf]

Supplementary Information 3. Univariate associations of pregnancy outcomes with vaginal *Candida* colonization (n=330[a]).

	n (%)	CC+ (%)	COR (95% CI)	p-value
Delivery features				
**Fundal height (cm)**				0.781
33–37	248 (75.1)	27.0	Ref.	
≤32	64 (19.4)	25.0	0.93 (0.58–1.48)	
≥ 38	18 (5.5)	33.3	1.23 (0.62–2.45)	
**Maternal temperature during labor**				0.055
<37.2°C	313 (94.9)	25.9	Ref.	
≥37.2°C	17 (5.1)	47.1	2.54 (0.95–6.82)	
**Labor induction**				0.836
Spontaneous	305 (92.4)	26.6	Ref.	
Misoprostol only	16 (4.9)	31.3	1.18 (0.56–2.49)	
Foley catheter + misoprostol	9 (2.7)	33.3	1.26 (0.49–3.23)	
**Duration of labor**				0.097
≤12h	306 (92.7)	28.1	Ref.	
>12h	24 (7.3)	12.5	0.44 (0.15–1.30)	
**Number of pelvic examinations during labor**				0.226
≤5	63 (19.1)	20.6	Ref.	
>5	267 (80.9)	28.5	1.38 (0.82–2.32)	
**Nurse's hand washing before delivery**				0.861
Yes	295 (89.4)	27.1	Ref.	
No	35 (10.6)	25.7	0.95 (0.52–1.72)	
**Utilization of sterile delivery kit**				0.671
Yes	239 (72.4)	27.6	Ref.	
No	91 (27.6)	25.3	0.92 (0.61–1.38)	
**Fetal presentation**				0.011
Cephalic	319 (96.7)	25.7	Ref.	
Non-cephalic	11 (3.3)	63.6	**5.05 (1.44–17.72)**	
**Type of delivery**				0.973
Vaginal delivery	274 (83.0)	27.0	Ref.	
Cesarean section	56 (17.0)	26.8	0.99 (0.62–1.60)	
**Neonatal eye disinfection with silver nitrate**				0.858
Yes	280 (84.9)	26.8	Ref.	
No	50 (15.1)	28.0	1.05 (0.64–1.70)	
**Cleaning umbilical cord**				0.685

None	314 (95.2)	26.8	Ref.	
Chlorhexidine	16 (4.8)	31.3	1.17 (0.55–2.47)	
Neonatal outcomes				
**Preterm birth[(b)]**				**0.024**
No	282 (85.5)	24.8	Ref.	
Yes	48 (14.5)	39.6	**1.59 (1.06 –2.39)**	
**PROM[(c)]**				0.739
Intact	262 (79.4)	26.0	Ref.	
Ruptured	68 (20.6)	27.9	1.08 (0.70–1.66)	
**Apgar at fifth minute**				0.650
>7	321 (97.3)	26.8	Ref.	
≤7	9 (2.7)	33.3	1.24 (0.48–3.19)	
**Sex of the newborn**				0.676
Female	168 (50.9)	25.9	Ref.	
Male	162 (49.1)	28.0	0.93 (0.65–1.32)	
**Visible deformity**				0.753
Absent	321 (97.3)	27.1	Ref.	
Present	9 (2.7)	22.2	0.82 (0.24–2.83)	
**Reported hypothermia**				
No	327 (99.1)	27.2	Ref.	
Yes	3 (0.9)	0.00	N/A	N/A
**Reported lethargy**				0.737
No	325 (98.5)	27.1	Ref.	
Yes	5 (1.5)	20.0	0.74 (0.13–4.31)	
**Reported jaundice**				
No	329 (99.7)	27.1	Ref.	
Yes	1(0.3)	0.0	N/A	N/A
**Reported seizure**				
No	330 (100)	27.0	Ref.	
Yes	0 (0.0)	N/A	N/A	N/A
**Reported hypotonia**				0.595
No	324 (98.2)	27.2	Ref.	
Yes	6 (1.2)	16.7	0.61 (0.10–3.72)	
**Reported hypertonia**				
No	330 (100)	27.0	Ref.	
Yes	0 (0.0)	N/A	N/A	N/A
**Difficult suction**				0.930
No	326 (98.8)	27.0	Ref.	
Yes	4 (1.2)	25.0	0.93 (0.17–5.12)	
**Dirty umbilicus**				

No	330 (100)	27.0	Ref.	
Yes	0 (0.0)	N/A	N/A	N/A
**Newborn feeding**				0.131
Breastfeeding	324 (98.2)	26.5	Ref.	
Mixed feeding	6 (1.8)	50.0	1.88 (0.83–4.28)	
**Neonatal temperature (°C)**				**0.013**
<36.6	171 (51.8)	25.7	1.01 (0.69–1.49)	
36.6–37.2	142 (43.0)	25.4	Ref.	
>37.2	17 (5.2)	52.9	**2.09 (1.23–3.55)**	
**Reported fever**				**<0.001**
No	326 (98.8)	26.4	Ref.	
Yes	4 (1.2)	75.0	**2.84 (1.56 –5.15)**	
**Neonatal height**				0.178
Normal (46–56 cm)	321 (97.3)	26.5	Ref.	
Short (<46 cm)	9 (2.7)	44.4	1.68 (0.79–3.57)	
**Weight at birth**[(c)]				0.813
≥2500g	313 (94.9)	26.8	Ref.	
<2500g	17 (5.1)	29.4	1.10 (0.51–2.34)	
**General physical state at birth**				0.406
Normal	322 (97.6)	27.3	Ref.	
Abnormal	8 (2.4)	12.5	0.46 (0.07–2.89)	
**Skin**				0.922
Normal	323 (97.9)	26.9	Ref.	
Abnormal	7 (2.1)	28.6	1.06 (0.32–3.48)	
**Mouth**				N/A
Normal	330 (100)	27.0	Ref.	
Abnormal	0 (0.0)	N/A	N/A	
**Ear and noise examination**				N/A
Normal	330 (100)	27.0	Ref.	
Abnormal	0 (0.0)	N/A	N/A	
**Neck**				N/A
Normal	330 (100)	27.0	Ref.	
Abnormal	0 (0.0)	N/A	N/A	
**Cardiovascular examination**[(d)]				0.341
Normal	321 (97.3)	27.4	Ref.	
Abnormal	9 (2.7)	11.1	0.41 (0.06–2.60)	
**Lung examination**[(e)]				0.531
Normal	319 (96.7)	27.3	Ref.	
Abnormal	11 (3.3)	18.2	0.67 (0.19–2.37)	
**Abdomen examination**				N/A

Normal	330 (100)	27.0	Ref.	
Abnormal	0 (0.0)	N/A	N/A	
**Cyanosis of extremities**				0.341
No	321 (97.3)	27.4	Ref.	
Yes	9 (2.7)	11.1	0.41 (0.06–2.60)	
**Neurological examination**				0.341
Normal	321 (97.3)	27.4	Ref.	
Abnormal	9 (2.7)	11.1	0.41 (0.06–2.60)	
**Genito-urinary examination**				0.488
Normal	323 (97.9)	27.2	Ref.	
Abnormal	7 (2.1)	14.3	0.52 (0.08–3.26)	
**NICU for suspicion of sepsis**				0.322
No	316 (95.8)	27.5	Ref	
Yes	14 (4.2)	14.3	0.52 (0.14–1.90)	

(a) Seven slides did not contain biological material or appeared damaged.
(b) Preterm birth was defined using WHO definitions (delivery at less than 37 completed weeks; less than 259 completed days (WHO, 1977)
(c) PROM: Premature rupture of membranes
(d) Low birth weight was defined as weight <2500gr (WHO, 1977)
(e) Bradycardia or tachycardia
(f) Abnormal if chest indrawing and respiratory rate >60 breaths per minute(b/m)
