# Supplementary material for: Second trimester vaginal Candida colonization among pregnant women attending antenatal care in Bukavu, Democratic Republic of the Congo: prevalence, clinical correlates, risk factors and pregnancy outcomes
Source: Front Glob Womens Health. 2024 May 23;5:1339821. doi: 10.3389/fgwh.2024.1339821 (PMC11153668; doi:10.3389/fgwh.2024.1339821)
Supplement: Supplementary file 4 [file Table4.pdf]

Supplementary Information 4. Univariate analysis of association between vaginal *Candida* concentration and PTB.

Candida concentration	n	PTB (%)	Crude OR (95% CI)	p-value
Negative	125	14 (11.2)	Ref.	
Low concentration	25	4 (16.0)	1.51 (0.45-5.04)	0.503
Moderate concentration	36	7 (19.4)	1.91 (0.71-5.18)	0.201
High concentration	16	5 (31.3)	3.60 (1.09-11.90)	0.035
