# Supplementary material for: Second trimester vaginal Candida colonization among pregnant women attending antenatal care in Bukavu, Democratic Republic of the Congo: prevalence, clinical correlates, risk factors and pregnancy outcomes
Source: Front Glob Womens Health. 2024 May 23;5:1339821. doi: 10.3389/fgwh.2024.1339821 (PMC11153668; doi:10.3389/fgwh.2024.1339821)
Supplement: Supplementary file 5 [file Table5.pdf]

Supplementary Information 5. Prevalence of vaginal *Candida* colonization among pregnant women in sub-Saharan Africa.

Country	Prevalence of *Candida*	Proportion *C albicans/Candida*	Population	Diagnostics	Reference
Burkina Faso	22.71%	40.4%	PW	GS, C	Sangaré et al., 2018
Burkina Faso	14%	NR	PW	WM	Meda et al., 1997
Cameroon	55.4%	NR	SPW	WM, GS, C	Toua et al., 2013
CAR	46.6%	NR	PW	WM, C	David Blankhart, Olaf Müller, Gérard Gresenguet, & Weis, 1999
**DRC**	**28.1%**	**91.0%**	**PW**	**GS**	**Current study**
Ethiopia	9.3%	NR	PW	WM, GS	Mulu, Yimer, Zenebe, & Abera, 2015
Gabon	30.8%	NR	PW	WM, GS	Bourgeois et al., 1998
Ghana	39.8%	NR	SPW	WM, C	Apea-Kubi, Sakyi, Yamaguchi, & Ofori-Adjei, 2005
Ghana	36.5%	NR	PW	WM	Konadu et al., 2019
Kenya	26.2%	NR	PW	WM	Thomas, Choudhri, Kariuki, & Moses, 1996
Kenya	55%	NR	PW	WM, GS	Fonck et al., 2000
Kenya	90.38%	63.8%	SPW	GS, C	Menza, Wanyoike, & Muturi, 2013
Kenya	23%	NR	PW	WM	Jespers et al., 2014
Mali	39%	NR	PW	WM, C	Mulanga-Kabeya et al., 1999
Mauritania	26%	NR	PW	GS, C	Sy et al., 2018
Nigeria	20.9%	NR	PW	C	Ekwempu, Lawande, & Egler, 1981
Nigeria	65%	NR	PW	C	Akerele, Abhulimen, & Okonofua, 2002
Nigeria	37.8%	NR	ASPW	WM, C	Aboyeji & Nwabuisi, 2003
Nigeria	56.3%	NR	PW	WM, C	Nwosu & Djieyep, 2007

Nigeria	26%	65.4%	PW	WM, C	Donbraye-Emmanuel et al., 2010
Nigeria	62.2%	NR	SPW	C	Akah, Nnamani, & Nnamani, 2010
Nigeria	30%	NR	PW	WM, C	Okonkwo & Umeanaeto, 2010
Nigeria	36%	NR	PW	WM, C	Olowe, Makanjuola, Olowe, & Adekanle, 2014
Nigeria	25%	60%	PW	WM, GS, C	Nurat, Babalola, Shittu, Tijani, & Adekola, 2015
Nigeria	60.76%	73.7%	SPW	WM, GS, C	Nnadi & Singh, 2017
Nigeria	45%	50.0%	PW[a]	GS, C	Mumuney & Abalaka, 2019
Nigeria	25%	69.2%	PW [b]	GS, C	Mumuney & Abalaka, 2019
Uganda	45%	78.95%	PW	WM, GS, C	Mukasa et al., 2015
South Africa	38.3%	70.27%	PW	WM, C	O'Farrell, Hoosen, Kharsany, & Van den Ende, 1989
South Africa	57%	NR	PW	WM	Jespers et al., 2014
Sudan	13.9%	NR	PW	WM	Ortashi, El Khidir, & Herieka, 2004
Sudan	16.6%	NR	PW	WM	Abdelaziz, Ibrahim, Bilal, & Hamid, 2014
Tanzania	65.6%	63.5%	SPW	C	Mushi, Mmole, & Mshana, 2019
Tanzania	NR	66.2%	PW	C	Namkinga, Matee, Kivaisi, Kullaya, & Mneney, 2005
Tanzania	11.4%	NR	PW	WM, GS	Msuya et al., 2009
Togo	49.7%	90.7%	SPW	WM, GS, C	Dakey, 2018
Togo	30.77%	NR	PW	WM, C	Tchelougou et al., 2013
Zimbabwe	39.9%	NR	PW	WM	Kurewa et al., 2010

[a]Ibrahim Badamasi Hospital, b General Hospital, Minna Niger State are the two centers selected for the study and results were presented separately.
SPW: symptomatic pregnant women attending antenatal care (ANC), PW: pregnant women attending ANC, C: culture-based detection and identification, GS: Gram-stain based microscopy, WM: wet mount microscopy, qPCR: quantitative PCR.

References.

- Abdelaziz ZA, Ibrahim ME, Bilal NE, Hamid ME. Vaginal infections among pregnant women at Omdurman Maternity Hospital in Khartoum, Sudan. The Journal of Infection in Developing Countries. 2014;8(04):490-7.
- Aboyeji A, Nwabuisi C. Prevalence of sexually transmitted diseases among pregnant women in Ilorin, Nigeria. Journal of Obstetrics and Gynaecology. 2003;23(6):637-9.
- Akah P, Nnamani C, Nnamani P. Prevalence and treatment outcome of vulvovaginal candidiasis in pregnancy in a rural community in Enugu State, Nigeria. Journal of Medicine and Medical Sciences. 2010;1(10):447-52.
- Akerele J, Abhulimen P, Okonofua F. Prevalence of asymptomatic genital infection among pregnant women in Benin City, Nigeria. African journal of reproductive health. 2002:93-7.
- Apea-Kubi KA, Sakyi B, Yamaguchi S, Ofori-Adjei D. Bacterial vaginosis, Candida albicans and Trichomonas vaginalis infection in antenatal and gynaecological patients in Ghana. Tropical Journal of Obstetrics and Gynaecology. 2005;22:108-12.
- Bourgeois A, Henzel D, Malonga-Mouelet G, Dibanga G, Tsobou C, Peeters M, et al. Clinical algorithms for the screening of pregnant women for STDs in Libreville, Gabon: which alternatives? Sexually transmitted infections. 1998;74(1):35-9.
- Blankhart D, Muller O, Gresenguet G, Weis P. Sexually transmitted infections in young pregnant women in Bangui, Central African Republic. International journal of STD & AIDS. 1999;10(9):609-14.
- Donbraye-Emmanuel O, Donbraye E, Okonko I, Alli J, Ojezele M, Nwanze J. Detection and prevalence of Candida among pregnant women in Ibadan, Nigeria. World Applied Science Journal. 2010;10(9):986-91.
- Dakey KA. Vaginal Infections in Pregnant Women at the Bè Hospital in Lomé (Togo) from 2008 to 2013. Open Access Library Journal. 2018;5(03):1.
- Ekwempu C, Lawande R, Egler L. Microbial flora of the lower genital tract of women in labour in Zaria, Nigeria. Journal of clinical pathology. 1981;34(1):82-3.
- Konadu DG, Owusu-Ofori A, Yidana Z, Boadu F, Iddrisu LF, Adu-Gyasi D, et al. Prevalence of vulvovaginal candidiasis, bacterial vaginosis and trichomoniasis in pregnant women attending antenatal clinic in the middle belt of Ghana. BMC pregnancy and childbirth. 2019;19(1):341.
- Fonck K, Kidula N, Jaoko W, Estambale B, Claeys P, Ndinya-Achola J, et al. Validity of the vaginal discharge algorithm among pregnant and non-pregnant women in Nairobi, Kenya. Sexually transmitted infections. 2000;76(1):33-8.
- Jespers V, Crucitti T, Menten J, Verhelst R, Mwaura M, Mandaliya K, et al. Prevalence and correlates of bacterial vaginosis in different sub-populations of women in sub-Saharan Africa: a cross-sectional study. PloS one. 2014;9(10):e109670.
- Kurewa NE, Mapingure MP, Munjoma MW, Chirenje MZ, Rusakaniko S, Stray-Pedersen B. The burden and risk factors of sexually transmitted infections and reproductive tract infections among pregnant women in Zimbabwe. BMC infectious diseases. 2010;10(1):127.
- Meda N, Sangare L, Lankoande S, Sanou PT, Compaore PI, Catraye J, et al. Pattern of sexually transmitted diseases among pregnant women in Burkina Faso, west Africa: potential for a clinical management based on simple approaches. Sexually Transmitted Infections. 1997;73(3):188-93.67
- Msuya SE, Uriyo J, Hussain A, Mbizvo EM, Jeansson S, Sam NE, et al. Prevalence of sexually transmitted infections among pregnant women with known HIV status in northern Tanzania. Reproductive health. 2009;6:4.
- Mukasa KJ, Herbert I, Daniel A, Sserunkuma KL, Joel B, Frederick B. Antifungal Susceptibility Patterns of Vulvovaginal Candida species among Women Attending Antenatal Clinic at Mbarara Regional Referral Hospital, South Western Uganda. British microbiology research journal. 2015;5(4):322-31.
- Mulanga-Kabeya C, Morel E, Patrel D, Delaporte E, Bougoudogo F, Maiga YI, et al. Prevalence and risk assessment for sexually transmitted infections in pregnant women and female sex workers in Mali: is syndromic approach suitable for screening? Sexually transmitted infections. 1999;75(5):358-9.
- Mulu W, Yimer M, Zenebe Y, Abera B. Common causes of vaginal infections and antibiotic susceptibility of aerobic bacterial isolates in women of reproductive age attending at Felegehiwot Referral Hospital, Ethiopia: a cross sectional study. BMC Womens Health. 2015;15:42.
- Mumuney KT, Abalaka ME, editors. Comparative Study of Vaginal Candidiasis in Pregnant and Non-Pregnant Women Attending Ibrahim Badamasi Babangida Specialist Hospital and General Hospital, Minna Niger State, Nigeria2019.
- Nwosu CO, Djieyep NA. Candidiasis and trichomoniasis among pregnant women in a rural community in the semi-arid zone, north-eastern Nigeria. West Afr J Med. 2007;26(1):17-9.

Okonkwo NJ, Umeanaeto P. Prevalence of Vaginal Candidiasis among Pregnant Women in Nnewi Town of Anambra State, Nigeria. African Research Review. 2011;4.

- Olowe OA, Makanjuola OB, Olowe RA, Adekanle DA. Prevalence of vulvovaginal candidiasis, trichomoniasis and bacterial vaginosis among pregnant women receiving antenatal care in Southwestern Nigeria. European journal of microbiology & immunology. 2014;4 4:193-7.
- O'Farrell N, Hoosen A, Kharsany A, Ende Jvd. Sexually transmitted pathogens in pregnant women in a rural South African community. Genitourinary Medicine. 1989;65:276 - 80.
- Ortashi OM, Khidir IE, Herieka E. Prevalence of HIV, syphilis, Chlamydia trachomatis, Neisseria gonorrhoea, Trichomonas vaginalis and candidiasis among pregnant women attending an antenatal clinic in Khartoum, Sudan. Journal of Obstetrics and Gynaecology. 2004;24:513-5.
- Nurat AA, Babalola GO, Shittu MO, Tijani MA, Adekola SA. Detection and Epidemiology of Vulvovaginal Candidiasis among Asymptomatic Pregnant Women Attending a Tertiary Hospital in Ogbomoso, Nigeria. International journal of biomedical research. 2015;6:518-23.
- Sangaré I, Sirima C, Bamba S, Zida A, Cisse M, Bazié WW, et al. Prevalence of vulvovaginal candidiasis in pregnancy at three health centers in Burkina Faso. Journal de mycologie medicale. 2018;28 1:186-92.
- Sy O, Diongue K, Ahmed CB, Ba O, Moulay F, Lo B, et al. [Vulvovaginal candidiasis in pregnant women in Nouakchott, Mauritania]. Journal de mycologie medicale. 2018;28 2:345-8.
- Tchelougou D, Karou DS, Kpotsra A, Balaka A, Assih M, Bamoké M, et al. [Vaginal infections in pregnant women at the Regional Hospital of Sokode (Togo) in 2010 and 2011]. Medecine et sante tropicales. 2013;23 1:49-54.
- Toua V, Djaouda M, Gaké B, Menye DE, Christie EA. Prevalence of Vulvovaginal Candidiasis amongst pregnant women in Maroua (Cameroon) and the sensitivity of Candida albicans to extracts of six locally used antifungal plants. International Research Journal of Microbiology. 2013;4:89-97.
- Thomas T, Choudhri S, Kariuki CH, Moses SP. Identifying cervical infection among pregnant women in Nairobi, Kenya: limitations of risk assessment and symptom-based approaches. Genitourinary Medicine. 1996;72:334 - 8.
- Nnadi DC, Singh S. The prevalence of genital Candida species among pregnant women attending antenatal clinic in a tertiary health center in North-west Nigeria. Sahel Medical Journal. 2017;20:33 - 7.
- Mukasa KJ, Herbert I, Daniel A, Sserunkuma KL, Joel B, Frederick B. Antifungal susceptibility patterns of vulvovaginal Candida species among women attending antenatal clinic at Mbarara Regional Referral Hospital, South Western Uganda. British microbiology research journal. 2015;5(4):322.68
